# Diagnostic performance of cardiac magnetic resonance segmental myocardial strain for detecting microvascular obstruction and late gadolinium enhancement in patients presenting after a ST-elevation myocardial infarction

**DOI:** 10.3389/fcvm.2022.909204

**Published:** 2022-07-14

**Authors:** Christoph Gräni, Anselm W. Stark, Kady Fischer, Monika Fürholz, Andreas Wahl, Sophie A. Erne, Adrian T. Huber, Dominik P. Guensch, René Vollenbroich, Andrea Ruberti, Stephan Dobner, Dik Heg, Stephan Windecker, Jonas Lanz, Thomas Pilgrim

**Affiliations:** ^1^Department of Cardiology, Inselspital, Bern University Hospital, University of Bern, Bern, Switzerland; ^2^Department of Anesthesiology and Pain Medicine, Inselspital, University Hospital Bern, University of Bern, Bern, Switzerland; ^3^Department of Diagnostic, Interventional and Pediatric Radiology, Inselspital, Bern University Hospital, University of Bern, Bern, Switzerland; ^4^Clinical Trials Unit, University of Bern, Bern, Switzerland

**Keywords:** STEMI, strain, feature tracking (CMR-FT), LGE CMR, microvascular obstruction (MVO), late gadolinium enhancement, myocardial strain analysis

## Abstract

**Background:**

Microvascular obstruction (MVO) and Late Gadolinium Enhancement (LGE) assessed in cardiac magnetic resonance (CMR) are associated with adverse outcome in patients with ST-elevation myocardial infarction (STEMI). Our aim was to analyze the diagnostic performance of segmental strain for the detection of MVO and LGE.

**Methods:**

Patients with anterior STEMI, who underwent additional CMR were enrolled in this sub-study of the CARE-AMI trial. Using CMR feature tracking (FT) segmental circumferential peak strain (SCS) was measured and the diagnostic performance of SCS to discriminate MVO and LGE was assessed in a derivation and validation cohort.

**Results:**

Forty-eight STEMI patients (62 ± 12 years old), 39 (81%) males, who underwent CMR (i.e., mean 3.0 ± 1.5 days) after primary percutaneous coronary intervention (PCI) were included. All patients presented with LGE and in 40 (83%) patients, MVO was additionally present. Segments in all patients were visually classified and 146 (19%) segments showed MVO (i.e., LGE+/MVO+), 308 (40%) segments showed LGE and no MVO (i.e., LGE+/MVO–), and 314 (41%) segments showed no LGE (i.e., LGE–). Diagnostic performance of SCS for detecting MVO segments (i.e., LGE+/MVO+ vs. LGE+/MVO–, and LGE–) showed an AUC = 0.764 and SCS cut-off value was –11.2%, resulting in a sensitivity of 78% and a specificity of 67% with a positive predictive value (PPV) of 30% and a negative predictive value (NPV) of 94% when tested in the validation group. For LGE segments (i.e., LGE+/MVO+ and LGE+/MVO– vs. LGE–) AUC = 0.848 and SCS with a cut-off value of –13.8% yielded to a sensitivity of 76%, specificity of 74%, PPV of 81%, and NPV of 70%.

**Conclusion:**

Segmental strain in STEMI patients was associated with good diagnostic performance for detection of MVO+ segments and very good diagnostic performance of LGE+ segments. Segmental strain may be useful as a potential contrast-free surrogate marker to improve early risk stratification in patients after primary PCI.

## Introduction

The presence of microvascular obstruction (MVO) in patients with ST-segment elevation myocardial infarction (STEMI) is a predictor of poor functional recovery, congestive heart failure and mortality (1, 2). Therefore, early detection of MVO is clinically relevant and helpful to guide adjunctive therapeutic decisions in patients with STEMI. MVO can be assessed non-invasively using cardiac magnetic resonance imaging (CMR) and is characterized by a no-flow phenomenon, with a dark appearance in the late gadolinium enhancement (LGE) images. However, in patients with poor renal function or intolerance to gadolinium contrast agent, the evaluation of MVO may potentially be limited. Furthermore, the impact of MVO on left ventricular myocardial function is largely unknown, and it remains to be determined, whether left ventricular myocardial function may act as a potential contrast-free surrogate marker for the presence of MVO. Myocardial deformation expressed as myocardial strain is an accurate parameter for the assessment of myocardial function and can depict ventricular function in different orientations (i.e., circumferential and longitudinal shortening) on a global and segmental level (3–6). In CMR, strain can be assessed with dedicated sequences like tissue tagging or with novel emerging post-processing software like CMR feature tracking (CMR-FT) (7). The aim of this study was to analyze segmental CMR-FT strain from routinely acquired ciné images and to test the diagnostic performance of segmental strain for the detection of MVO and LGE in STEMI patients after primary percutaneous coronary intervention.

## Materials and methods

### Participants

**FIGURE 1:**
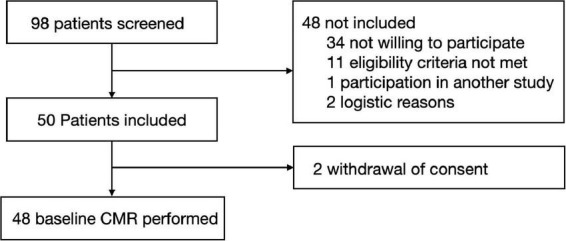
Flowchart. The flowchart illustrates screening, withdrawals, and performed CMR analysis.

The participants of this study were enrolled in the CARE-AMI trial (clinical trial unique identifier: NCT03274752) (8). Detailed in- and exclusion criteria have been reported previously. In brief, patients presenting with anterior STEMI and reduced LVEF (i.e., ≤ 45%), who underwent primary percutaneous coronary intervention (PCI) within 24 h were eligible for inclusion and underwent baseline CMR after PCI (8). Exclusion criteria consisted of previous myocardial infarction (MI) or primary percutaneous intervention along with contraindications to CMR (8) (see Figure 1).

### Cardiac magnetic resonance protocol

Scans were performed on a 1.5 Tesla system (Magnetom Aera, Siemens Healthineers, Erlangen, Germany). Standard steady state-free precession 8 mm slice thickness ciné images were acquired covering the left and right ventricle in a short-axis (SAX) stack without gap and as well three long axis (LAX) views were obtained to cover for the whole ventricle to assess ventricular function and dimensions. For the LGE images, patients received gadobutrol as a contrast agent (0.2 mmol/kg) and 10–15 min post injection of contrast agent LGE images were acquired as described elsewhere (9).

### Tissue characterization

All CMR data were read by readers blinded to patient information and coronary angiography findings. Semi-quantitative LGE images were analyzed by the five standard deviation approach for %-of enhanced myocardial mass (10). MVO was defined as an endocardially located no-reflow region represented by a black area with surrounding LGE. Segments were visually classified as segments with LGE and additional MVO (LGE+/MVO+), segments with LGE and no MVO (LGE+/MVO–) or segments with no enhancement (LGE–) (9).

### Strain analysis

CMR-FT, a post-processing technique was applied to standard steady state-free precession cinés in which with the help of epi- and endocardial contours features within the myocardium were tracked over the entire cardiac cycle to obtain myocardial strain (7). CMR-FT analysis was performed using Circle Cardiovascular Imaging (cvi^42^, Calgary, Canada, version 5.12) software. End diastolic, epi-, and endocardial contours were placed using the automatic contouring tool on a SAX stack, and three LAX (4-chamber, 3-chamber, and 2-chamber) ciné images, and adjusted manually by the reader if necessary. Strain was calculated for systolic deformation parameters represented by peak strain (point of maximum deformation), time to peak strain (within the cardiac cycle), along with systolic strain rate (change of strain over time) in both the circumferential and longitudinal orientations (i.e., segmental circumferential peak strain, SCS; segmental longitudinal peak strain, SLS) (5, 7, 11). Measurements were reported per American heart association (AHA) segment (16 segment model). In our experience, the average time for automated strain analysis with data reporting takes about 5 min resulting in global and segmental strain values simultaneously. However, due to inconsistencies in the segmental strain analysis (i.e., highest peak at incorrect phase) manual corrections can take up to 20 min per patient.

### Statistical analysis

Continuous variables were expressed as mean ± standard deviation unless defined otherwise or median and interquartile range based on normality. Categorical variables were presented as frequency and percent of the population. Statistical significance was defined as a two-tailed *p*-value < 0.05. Patient characteristics and global findings are reported for the entire cohort, as well as grouped by the presence or absence of MVO. These groups were compared by a Chi-square test or an independent *t*-test, for categorical or continuous variables, respectively.

First, to characterize segmental CMR-FT measurements based on the presentation of co-localized MVO or LGE, segmental strain values were categorized into the three groups LGE+/MVO+, LGE+/MVO–, and LGE–. To compare these three groups, a mixed linear model was fitted with the *lmer* and *emmeans* (6, 12) functions accounting for multiple measurements per patient by including subject identification as a random intercept and reported as mean and standard error.

The diagnostic performance was initially tested using logistic regression to investigate the association of sex, laboratory markers, coronary angiography findings and all regional CMR-FT measurements to the presence of MVO. Specifically for peak circumferential and longitudinal strain measures, further diagnostic analysis was conducted. Patients were randomized into a derivation group of 30 patients (480 segments) and a validation group of 18 patients (288 segments). In the derivation group, receiver-operator characteristic (ROC) curves were created to test the ability of strain measures to first discriminate between scar and no scar (LGE+ vs. LGE–), MVO and no MVO (MVO+ vs. MVO–) and for MVO discrimination within scar (LGE+/MVO+ vs. LGE+/MVO–). ROC curve analysis for peak strain was performed in a derivation cohort and then tested in a validation cohort. Optimal cut-off values for the different ROC curves were calculated with the Youden Index using the pROC function (13). These cut-offs were subsequently tested for performance in the validation cohort, with the 95% confidence intervals of sensitivity, specificity, negative predictive value, and positive predictive value corrected for the multiple measures per patient (14).

Images of 10 randomly selected participants were replicated by a second independent reader and inter-observer reliability of one segment was assessed using a two-way intraclass correlation (ICC) test for absolute measures. Statistical analyses were performed with R software (version 3.5.0, R Foundation for Statistical Computing, Vienna, Austria), GraphPad Prism version 9.0 (GraphPad Software, La Jolla California United States) with SPSS version 24 (SPSS IBM, New York, United States).

## Results

### Patient demographics

Fifty patients with anterior STEMI undergoing primary PCI were enrolled between 10/2017, and 09/2020. After withdrawal of 2 patients, 48 patients (62 ± 12 years) remained for the purpose of the present analysis (96%). Thirty-nine (81%) patients were male. CMR exams were performed during the hospital admission within a mean of 3.0 ± 1.5 days after PCI. A total of 722 segments of 753 segments (96%) for short axis slices could be analyzed and 653 of 768 segments (89%) for longitudinal orientation. Detailed demographics for patient’s characteristics are depicted in Table 1. On a patient level, LGE was present in all patients and 40 (83%) patients showed additionally presence of MVO. LVEF of all patients was 40 ± 9%, while LVEF was significantly lower in patients with MVO+ when compared to MVO– patients (39 ± 9% vs. 48 ± 8%; *p* = 0.013) (Table 1).

**TABLE 1:** Baseline characteristics.

Demographics	Total population (*n* = 48)	MVO+ (*n* = 40)	MVO– (*n* = 8)	*P*-value
Age	61.8 ± 12.5	60.4 ± 12.8	66.9 ± 8.3	*p* = 0.090
Sex (male)	39 (81%)	34 (85%)	5 (63%)	*p* = 0.137
BMI	27.2 ± 4.6	27.9 ± 4.7	25.2 ± 1.8	*p* = 0.010
Time to Balloon (min)	290 (149–638.5)	215 (133–833)	312 (150–633)	*p* = 0.785
Killip I (no clinical signs or symptoms of heart failure)	33 (69%)	26 (65%)	7 (88%)	*p* = 0.210
Killip II (3rd heart Sound, rales and radiographic evidence of heart failure)	7 (15%)	6 (15%)	1 (13%)	*p* = 0.855
Killip III (pulmonary edema)	3 (6%)	3 (8%)	0	NA
Killip IV (cardiogenic shock)	5 (10%)	5 (13%)	0	NA
**Laboratory values**				
CK-MB	325 (205–559)	370 (252–584)	150 (93–271)	*p* = 0.098*
Troponin T	7,303 (4,533–13,192)	7,788 (6,128–13,922)	3,572 (2,402–4621)	*p* = 0.584*
NT-proBNP	1,529 (320–3,222)	1,529 (320–3,222)	1,211 (330–3,159)	*p* = 0.784*
**Medication**				
ACE inhibitor/AT II antagonist	48 (100%)	40 (100%)	8 (100%)	NA
Beta blocker	48 (100%)	40 (100%)	8 (100%)	NA
Aldosterone antagonist	20 (42%)	19 (48%)	1 (13%)	*p* = 0.067
SGLT-2 inhibitor	7 (15%)	6 (15%)	1 (13%)	*p* = 0.855
**Comorbidities**				
Family history	11 (23%)	11 (28%)	0	NA
Smoking current and former	19 (40%)	18 (45%)	1 (13%)	*p* = 0.086
Hypertension	22 (46%)	18 (45%)	4 (50%)	*p* = 0.796
Hypercholesterolemia	20 (42%)	17 (43)	3 (38%)	*p* = 0.793
**CMR measurements**				
LVEF	40.3 ± 9.1	38.8 ± 8.7	47.6 ± 7.5	*p* = 0.013
Total LGE extent (%)	37.1 ± 12.6	38.9 ± 11.7	27.9 ± 13.7	*p* = 0.064
Segments with LGE+/MVO–	308 (40%)	252 (39%)	56 (44%)	*p* = 0.16
Segments with LGE+/MVO+	146 (19%)	146 (23%)	0	NA
Segments with LGE–	314 (41%)	242 (38%)	72 (56%)	*p* = 0.14
**Coronary angiography measurements**				
Number of complete LAD occlusion	38 (79%)	33 (83%)	5 (63%)	*p* = 0.204
1 vessel disease	23 (48%)	20 (50%)	3 (38%)	*p* = 0.518
2 vessel disease	15 (31%)	11 (28%)	4 (50%)	*p* = 0.210
3 vessel disease	10 (21%)	9 (23%)	1 (13%)	*p* = 0.525

*Data was normalized using logarithmic scaling and compared using student’s t-test. Baseline demographics between patients with MVO (MVO+) and patients without MVO (MVO–). Groups were compared by a Chi-square test or an independent t-test, for categorical or continuous variables, respectively.

### Strain measurements

**FIGURE 2:**
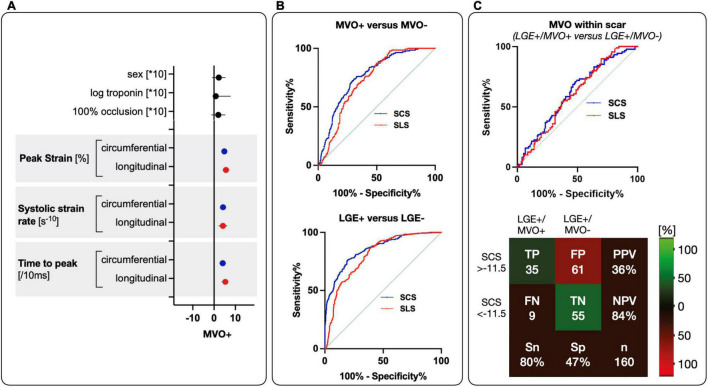
Diagnostic performance of SCS and SLS for MVO and LGE. **(A)** Parameter estimates with 95% confidence intervals demonstrate association with MVO presence. Blue for circumferential values and red for longitudinal values. **(B)** On top show ROC curves segmental circumferential and longitudinal peak strain for the detection of MVO+ (LGE+/MVO+) against MVO– (LGE+/MVO– and LGE–) (SCS with AUC = 0.764, *p* < 0.001 and a cut-off value of –11.2%, SLS with AUC = 0.728, *p* < 0.001 and a cut-off of –11.5%). On the bottom is segmental circumferential and longitudinal peak strain for detection of LGE+ (LGE+/MVO+, and LGE+/MVO–) against LGE– (SCS with AUC = 0.848, *p* < 0.001 and cut-off of –13.8%, SLS with AUC = 0.806, *p* < 0.001 and cut-off of –13.5%). **(C)** Diagnostic performance for MVO discrimination within scar (LGE+/MVO+ vs. LGE+/MVO–) with ROC curves of SCS and SLS from a derivation group on top. On the bottom is the performance of the cut-off from the ROC curve in a validation cohort with corresponding true positive (TP), false positive (FP), false negative (FN), true negative (TN), sensitivity (Sn), specificity (Sp), PPV, and NPV.

On a segmental level, 146 (19%) segments were categorized as LGE+/MVO+, 308 segments (40%) as LGE+/MVO–, and 314 (41%) segments as LGE–. SCS significantly differed between all categories, and was significantly impaired in LGE+/MVO+ segments (–9.0 ± 0.6%) in comparison to LGE+/MVO– (–10.4 ± 0.5%), and LGE– (–17.4 ± 0.5%) (*p* ≤ 0.007, Table 2). Similar results were observed for circumferential systolic strain rate and less prominent with time to peak. SLS was also poorest in LGE+/MVO+ segments (–6.6 ± 0.7%), followed by LGE+/MVO– (–8.3 ± 0.5%), while LGE– segments yielded the best SLS (–16.0 ± 0.5%, *p* ≤ 0.020 for all comparisons). With segmental longitudinal time to peak strain and systolic strain rate, only LGE– territories differed from LGE+/MVO+ (Table 2). Logistic regression demonstrated that in addition to segmental peak strain, time to peak strain, and systolic strain rate in both orientations were associated with the presence of MVO (Figure 2).

**TABLE 2:** Comparison of segmental strain between LGE– segments vs. LGE+/MVO– segments, vs. LGE+/MVO+ segments.

	LGE– (314 segments)	LGE+/MVO– (308 segments)	LGE+/MVO+ (146 segments)	*P*-value (LGE– vs. LGE+/MVO–)	*P*-value (LGE– vs. LGE+/MVO+)	*P*-value (LGE+/MVO– vs. LGE+/MVO+)
**Segmental LV strain**						
**Peak strain (%)**						
Circumferential	–17.4 ± 0.5	–10.4 ± 0.5	–9.0 ± 0.6	<0.001	<0.001	0.007
Longitudinal	–16.0 ± 0.5	–8.3 ± 0.5	–6.6 ± 0.7	<0.001	<0.001	0.020
**Time to peak strain (ms)**						
Circumferential	295 ± 6.4	339 ± 6.4	359 ± 7.9	<0.001	<0.001	0.007
Longitudinal	315 ± 6.9	366 ± 6.9	373 ± 9.1	<0.001	<0.001	0.502
**Systolic strain rate (/s)**						
Circumferential	–1.09 ± 0.04	–0.51 ± 0.04	–0.38 ± 0.06	<0.001	<0.001	0.032
Longitudinal	–1.09 ± 0.06	–0.55 ± 0.06	–0.40 ± 0.09	<0.001	<0.001	0.139

Comparison between groups with a mixed linear model accounting for multiple measurements per patient by including subject identification as a random intercept and reported as mean and standard error.

### Diagnostic performance

For scar (LGE+/MVO+ and LGE+/MVO–) vs. no scar (LGE–), ROC curve analysis of SCS showed an area under the curve (AUC) of 0.848 with an optimal cut-off of –13.8%. For SLS AUC was 0.806 and the cut-off was calculated at –13.5% (comparison between AUC *p* = 0.017). Application of these cut-offs in the validation group for SCS yielded a sensitivity of 76%, a specificity of 74%, a positive predictive value (PPV) of 81% and a negative predictive value (NPV) of 70%. SLS yielded to a sensitivity of 88%, a specificity of 65%, a PPV of 73%, and a NPV of 71%.

For MVO (LGE+/MVO+) vs. no MVO (LGE+/MVO– and LGE–) ROC curve analysis of SCS showed an AUC of 0.764 with an optimal cut-off of –11.2%. SLS showed an AUC of 0.728 with an optimal cut-off of –11.5% (comparison between AUC *p* = 0.020).

When applying these cut-offs to the validation cohort, the cut-off of –11.2% for SCS yielded a sensitivity of 78%, a specificity of 67%, a NPV of 94% and a PPV of 30%. For LGE+, application of the SLS cut-off of –11.5%, yielded a sensitivity of 80%, a specificity of 51%, a NPV of 92% and a PPV of 24%.

For MVO discrimination within scar (LGE+/MVO+ vs. LGE+/MVO–) the ROC curve analysis for SCS showed an AUC of 0.606 with an optimal cut-off of –11.5%. For SLS AUC was 0.612 with an optimal cut-off of –11.4% (comparison between AUC *p* = 0.323).

**FIGURE 3:**
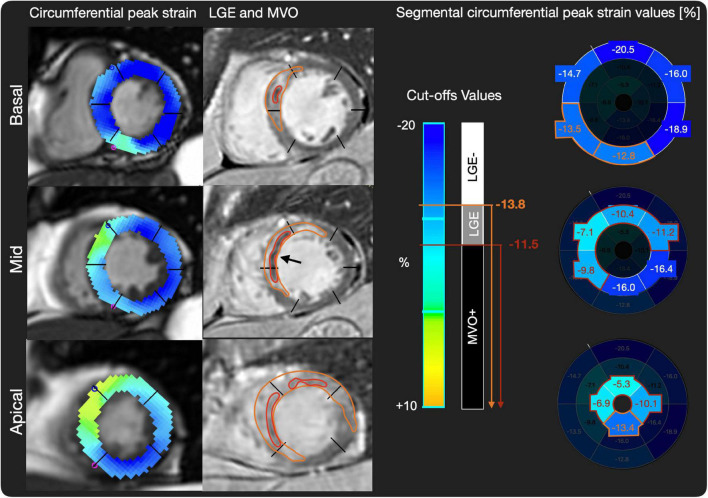
Patient example of LGE, MVO, and SCS values. On the left side, short axis basal, midventricular, and apical slices of circumferential strain images are shown. On the middle row matching late gadolinium enhancement (LGE) images with scar tissue in white (orange region of interest) and microvascular obstruction (MVO) (black arrow and red region of interest) are shown. On the right side, cut-off values for LGE+/MVO–, and MVO+, respectively, are shown and exemplary peak strain values for AHA segments are shown, where predicted LGE segments are highlighted in orange and predicted MVO segments in red.

Application of these cut-offs in the validation cohort for SCS yielded to a sensitivity of 80%, a specificity of 47%, a NPV of 84% and a PPV of 36%. For SLS the cut-off application in the validation cohort yielded to a sensitivity of 80%, a specificity of 31%, a NPV of 83%, and a PPV of 39% (Table 3). A patient examle for the application of the cut-offs can be seen in Figure 3.

**TABLE 3:** Diagnostic performance of segmental strain.

Parameter	Cut-off value	Sensitivity (%)	Specificity (%)	PPV (%)	NPV (%)	*P*-value
**Scar** vs. **no scar** **LGE+ vs. LGE**– (*i.e., LGE+/MVO+ and LGE+/MVO– vs. LGE–*)						
**Peak strain (%)**						
Circumferential	–13.8	76 (68–86)	74 (61–86)	81 (74–86)	70 (61–77)	<0.001
Longitudinal	–13.5	88 (66–95)	65 (56–87)	73 (63–83)	71 (62–80)	<0.001
**MVO** vs. **no MVO** *(i.e., LGE+/MVO+ vs. LGE+/MVO–)*						
**Peak strain (%)**						
Circumferential	–11.2	78 (64–92)	67 (59–76)	30 (22–38)	94 (89–97)	<0.001
Longitudinal	–11.5	80 (67–94)	51 (45–56)	24 (17–31)	92 (86–96)	<0.001
**MVO within scar** *(i.e., LGE+/MVO+ vs. LGE+/MVO–)*						
**Peak strain (%)**						
Circumferential	–11.5	80 (65–94)	47 (39–56)	36 (30–41)	84 (76–92)	<0.001
Longitudinal	–11.4	84 (76–92)	57 (47–66)	39 (36–41)	83 (77–90)	<0.001

Optimal cut-off values for the different ROC curves were calculated with the Youden Index. These cut-offs were subsequently tested for performance in the validation cohort, with the 95% confidence intervals of sensitivity, specificity, negative predictive value, and positive predictive value corrected for the multiple measures per patient.

### Reproducibility

The intraclass correlation coefficient for intrareader variability was 0.869 (95% CI: 0.800–0.912, *p* < 0.001) for SCS and 0.779 (95% CI: 0.697–0.839, *p* < 0.001) for SLS.

For interreader variability the values were 0.665 (95% CI: 0.514–0.768, *p* < 0.001) for SCS and 0.765 (95% CI: 0.685–0.827, *p* < 0.001) for the SLS. With regard to the inter- and intrareader variability for LGE mass (5SD method), we refer to a recent publication by our group, where the technique has proven to be a robust method (9).

## Discussion

The current study demonstrates that segmental strain in STEMI patients showed a good diagnostic performance for detecting MVO+ segments and very good diagnostic performance for LGE+ segments. However, the accuracy for discriminating MVO+ segments within LGE+ segments is limited.

In patients presenting after STEMI the use of CMR allows not only the depiction of left ventricular dimensions and ejection fraction, but also infarct size, MVO and myocardial strain. As MVO can only be detected by using gadolinium contrast, agent potentially limiting the performance in patients with poor renal function, intolerance to contrast agent or inability to undergo long scanning times, a non-contrast method would be desirable for indirect MVO detection. In line with previous studies, our study adds to the growing body of evidence that myocardial strain is correlated with LGE and MVO. In fact, this is the first study to the best of our knowledge, using CMR-FT from routinely acquired ciné images to analyze segmental strain for MVO detection. Previous studies have reported a correlation of strain with the presence of LGE in STEMI patients (15, 16) and MVO in addition to LGE seems to have a greater effect on strain (i.e., global) compared to LGE alone (17, 18). Furthermore, it has been demonstrated that segmental strain bears the potential to detect MVO with high accuracy, however, specific cut-off values vary between groups due to different patient cohorts and the use of different modalities (i.e., echocardiographic strain or tagging sequences). In fact, Everaars et al. (18) analyzed segmental circumferential peak strain with tissue tagging and found that a cut-off value of –6.2% could detect MVO with comparable sensitivity and specificity and high NPV and low PPV. Furthermore, CMR-FT over CMR tissue tagging inherits the advantage that it can be performed post-scanning with no need for a dedicated sequence and no prolongation of scan times, which decreases cost and increases efficiency and patient comfort. Bergerot et al. (19) investigated segmental longitudinal peak strain as assessed with a different modality (i.e., by speckle tracking echocardiography) and found that a SLS of –12.5% predicted MVO with high sensitivity and specificity (sensitivity of 85% specificity of 75%). However, speckle tracking echocardiography might be challenged by possible poor acoustic windows. The application of post-processing CMR-FT ciné images is rapidly expanding and Polacin et al. (20) were the first to evaluate a relevant cut-off for the detection of LGE with an optimal cut-off of –7.2% (sensitivity 89% specificity 86%). Compared to our study, the authors analyzed a different cohort. They included only patients with chronic infarctions, while in our study patients with acute anterior STEMI were analyzed. Consequently, in our analysis LGE might represent not only scar, but also edema, as scans were obtained within a short period of time after the event. Similarly, Yu et al. (15) found in a patient cohort scanned within 1 month after the infarction a SCS cut-off of –10.2%, assessed by CMR-FT that yielded LGE detection with a sensitivity of 80% and specificity of 85%. When comparing different techniques (i.e., CMR-FT and TT) in patients after STEMI, TT showed a higher interobserver variability and correlated less strongly with infarct and showed overall higher values for circumferential strain (21, 22), a possible factor contributing to different cut-off values and differences in the diagnostic performances. The same holds true for the comparison between echocardiography and CMR-FT with comparable results, however, not completely interchangeable (23–25). Regarding the differences between SCS and SLS, the literature is inconclusive, showing no superiority of either one (26–28). We would explain the higher diagnostic performance in our cohort mainly due to a higher robustness to outliers/wrong values. SLS is assessed in 3 long axis slices, every segment is depicted exactly once, while in a SAX stack every segment is depicted at least twice and results are averaged over the segment, as we also observed in our myocarditis cohort (6). This could also be different in patients with smaller infarct sizes, with only endocardial layer affected, where SLS could be more important. As we included only patients with acute anterior infarctions, infarct size may also have affected the strain in remote territory—another factor for differences in cut-off values. This is in line with the findings of a recent study by Sjögren et al. were the comparison of strain in different culprit lesions, infarctions in the territory of the LAD affected remote strain (29). The most likely explanation for this phenomenon is related to tethering of strain from remote segments next to infarct, that are more affected compared to non-neighboring segments (15, 30, 31). The same seems to hold true for LGE segments adjacent to MVO segments as in our population, where “borderline” zones might have altered the calculated cut-off values. LGE extent results may differ depending on the selected semi-quantitative post-processing LGE definition. The 5SD approach shows a low inter- and intrareader reliability (10, 32) and seems to represent an accurate method to assess scar in acute myocardial infarction (33). For segmental LGE and MVO analysis we decided to stick to a visual assessment for this analysis, but this would be a topic of interest in the future.

Previous studies and ours support the findings that CMR-FT shows the potential to accurately discriminate LGE segments and presence of MVO segments and may therefore help to better risk stratify patients with anterior STEMI after primary PCI. Diagnostic performance testing in future studies with larger cohorts could further help to establish cut-off values required before implementation in routine clinical practice.

### Study limitations

There are several limitations in this study. First, this was a single center study with a rather small sample size and no pre-specified power analysis for the diagnostic performance of myocardial strain assessed by CMR. Second, since segmental strain analysis is still a method in its early stages, we considered the CARE-AMI dataset with a homogenous cohort and only anterior infarction an ideal study population for the proof of concept analysis of segmental strain for detection of LGE and MVO in STEMI patients. Future studies should aim at reproducing data in cohorts with smaller infarctions and in other vessel territories. Currently, there are also no established normal values for segmental strain, however, there are some small cohort studies, where segmental strain was evaluated in healthy volunteers or in other cardiac pathologies (26, 27). Third, most patients were MVO+ which resulted in an uneven distribution; however, on a segmental level, the distribution between LGE+/MVO– vs. LGE+/MVO+, and LGE– was more evenly distributed, but may still represent a confounding factor. Definition of segments with LGE should also be evaluated through the use of different quantification techniques other than 5SD approach. Fourth, as there were no CMR sequences performed for the evaluation of edema or hemorrhage, the tissue damage is limited to the LGE+ segments, which may lead to an overestimation of remote territory. Fifth, through the tethering effect of segmental strain, adjacent segments of remote myocardium may further influence cut-off values, especially in cases with large infarcts. Finally, the CMR-FT method might be limited by a high variability compared to other techniques as observed by other groups (34, 35). Beside tagging, regional (fast) strain-encoded magnetic resonance (SENC) is a reliable method to image myocardial strain and inherits the potential to predict ischemia, size and transmurality of infarction (36–40). An important limitation of the current study is the lack of follow-up data, including the assessment of functional recovery and outcomes on a patient-level (40).

## Conclusion

Segmental strain analysis by CMR feature tracking is able to discriminate segments with MVO and/or LGE in patients with acute anterior STEMI undergoing primary PCI with good diagnostic accuracy. However, the accuracy for the discrimination of MVO presence within infarcted segments only, is limited. Segmental strain may be useful as a potential contrast-free surrogate marker to improve early risk stratification in patients after primary PCI.

## Data availability statement

The raw data supporting the conclusions of this article will be made available by the authors, without undue reservation.

## Ethics statement

The participants of this study were enrolled in the CARE-AMI trial (clinical trial unique identifier: NCT03274752). KEK Bern, Switzerland approved the study. The patients/participants provided their written informed consent to participate in this study.

## Author contributions

CG, AS, and KF analyzed the data and wrote the first draft. MF, AW, SE, AH, DG, RV, AR, SD, DH, SW, JL, and TP involved in acquiring the data from invasive coronary angiography, cardiac magnetic resonance imaging, post-processing, and contributing to writing the manuscript. All authors contributed to the article and approved the submitted version.

## Conflict of Interest

TP reported research grants to his institution from Edwards Lifesciences, Boston Scientific, and Biotronik; personal fees from Biotronik and Boston Scientific as a speaker and HighLife SAS for clinical event committee participation; and proctoring with Medtronic and Boston Scientific outside of this study. SW reported research and educational grants to his institution from Abbott, Amgen, AstraZeneca, BMS, Bayer, Boston Scientific, Biotronik, Cardinal Health, CardioValve, CSL Behring, Daiichi Sankyo, Edwards Lifesciences, Guerbet, InfraRedx, Johnson and Johnson, Medicure, Medtronic, Novartis, Pfizer, Polares, OrPha Suisse, Regeneron, Sanofi Aventis, Terumo, Sinomed, and V-Wave. SW served as unpaid advisory board member and/or unpaid member of the steering/executive group of trials funded by Abbott, Abiomed, Amgen, Astra Zeneca, Bayer, BMS, Boston Scientific, Biotronik, Cardiovalve, Edwards Lifesciences, Janssen, MedAlliance, Medtronic, Novartis, Polares, Recardio, Sinomed, Terumo, V-Wave and Xeltis, but has not received personal payments by pharmaceutical companies or device manufacturers. He was also member of the steering/executive committee group of several investigator-initiated trials that receive funding by industry without impact on his personal remuneration. RV reported grants from Clinical Trials Unit of the University of Bern during the conduct of the study. CG reported receiving funding from the Swiss National Science Foundation. DH reported affiliations with the Clinical Trials Unit Bern of the University of Bern, which has a staff policy of not accepting honoraria or consultancy fees; Clinical Trials Unit Bern is involved in design, conduct, or analysis of clinical studies funded by not-for-profit and for-profit organizations, and pharmaceutical and medical device companies provide direct funding to some of these studies. An up-to-date list of Clinical Trials Unit Bern’s conflicts of interest is at http://www.ctu.unibe.ch/research/declaration_of_interest/index_eng.html. SD reported research grants to the institution from Pfizer. JL reported grants from Bangerter-Rhyner Foundation and grants from University of Bern during the conduct of the study. The remaining authors declare that the research was conducted in the absence of any commercial or financial relationships that could be construed as a potential conflict of interest.

## Publisher’s note

All claims expressed in this article are solely those of the authors and do not necessarily represent those of their affiliated organizations, or those of the publisher, the editors and the reviewers. Any product that may be evaluated in this article, or claim that may be made by its manufacturer, is not guaranteed or endorsed by the publisher.

## Funding

This study was supported by dedicated grants from the Clinical Trial Unit of the University of Bern and the Bangerter-Rhyner Foundation.

## Acknowledgments

We thank Laura Morf, Nicole Reusser, and Lukas Lüthi from the research study team for their excellent technical and administrative support.

## Supplementary material

The Supplementary Material for this article can be found online at: https://www.frontiersin.org/articles/10.3389/fcvm.2022.909204/full#supplementary-material

Supplementary Figure 1 ICC Bland-Altman plots. Top row: Interreader variability of SLS on the left and SCS on the right, respectively, with 95% confidence interval for the levels of agreement. Bottom row: Intrareader reliability of SLS on the left and SCS on the right with 95% confidence interval for the levels of agreement.

Supplementary Figure 2 Confusion matrices for SCS and SLS for tissue discrimination. On the left: Confusion matrix showing the discrimination between LGE+/MVO+ (2), LGE+/MVO– (1) and LGE– (0) in a validation cohort with the use of cut-offs for SCS of –13.8 and –11.2%. Prediction results in a big FP rate for LGE+/MVO+, as can be seen in comparison of the row sums against column sums. On the right: The same can be seen for performance of SLS in a validation cohort with the cut-offs of –13.5 and –11.5%, which also result in a high amount of FP values for LGE+/MVO+. Of note: A perfect confusion matrix only shows results for the diagonal.

## Abbreviations

AUC: Area under the curve
CMR: Cardiovascular magnetic resonance imaging
FT: Feature Tracking
LAX: Long axis
LGE: Late gadolinium enhancement
LVEF: Left ventricular ejection fraction
MI: Myocardial infarction
MVO: Microvascular obstruction
NPV: Negative predictive value
PPV: Positive predictive value
ROC: Receiver-operator characteristic
SAX: Short axis
SCS: Segmental circumferential peak strain
SLS: Segmental longitudinal peak strain
STE: Speckle tracking echocardiography
STEMI: ST-elevation myocardial infarction
TT: Tissue tagging.
