# Supplementary figures and images for: Diagnostic performance of cardiac magnetic resonance segmental myocardial strain for detecting microvascular obstruction and late gadolinium enhancement in patients presenting after a ST-elevation myocardial infarction
Source: Front Cardiovasc Med. 2022 Jul 14;9:909204. doi: 10.3389/fcvm.2022.909204 (PMC9329615; doi:10.3389/fcvm.2022.909204)

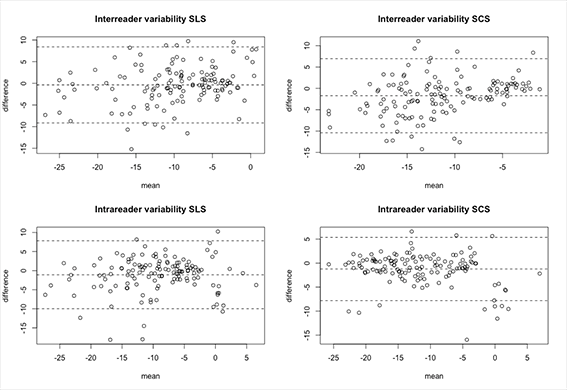

Supplement: Supplementary Figure 1 — ICC Bland-Altman plots. Top row: Interreader variability of SLS on the left and SCS on the right, respectively, with 95% confidence interval for the levels of agreement. Bottom row: Intrareader reliability of SLS on the left and SCS on the right with 95% confidence interval for the levels of agreement. [file Image_1.tif]

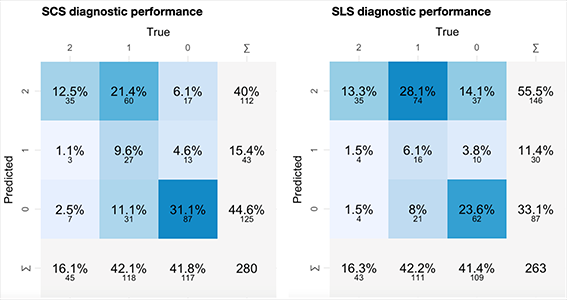

Supplement: Supplementary Figure 2 — Confusion matrices for SCS and SLS for tissue discrimination. On the left: Confusion matrix showing the discrimination between LGE+/MVO+ (2), LGE+/MVO– (1) and LGE– (0) in a validation cohort with the use of cut-offs for SCS of –13.8 and –11.2%. Prediction results in a big FP rate for LGE+/MVO+, as can be seen in comparison of the row sums against column sums. On the right: The same can be seen for performance of SLS in a validation cohort with the cut-offs of –13.5 and –11.5%, which also result in a high amount of FP values for LGE+/MVO+. Of note: A perfect confusion matrix only shows results for the diagonal. [file Image_2.tif]
